# Asymmetric Transfer of Auditory Perceptual Learning

**DOI:** 10.3389/fpsyg.2012.00508

**Published:** 2012-11-20

**Authors:** Sygal Amitay, Yu-Xuan Zhang, David R. Moore

**Affiliations:** ^1^Medical Research Council – Institute of Hearing ResearchNottingham, UK

**Keywords:** perceptual learning, transfer of learning, frequency discrimination, internal noise, phase locking, integration time, auditory, modeling

## Abstract

Perceptual skills can improve dramatically even with minimal practice. A major and practical benefit of learning, however, is in transferring the improvement on the trained task to untrained tasks or stimuli, yet the mechanisms underlying this process are still poorly understood. Reduction of internal noise has been proposed as a mechanism of perceptual learning, and while we have evidence that frequency discrimination (FD) learning is due to a reduction of internal noise, the source of that noise was not determined. In this study, we examined whether reducing the noise associated with neural phase locking to tones can explain the observed improvement in behavioral thresholds. We compared FD training between two tone durations (15 and 100 ms) that straddled the temporal integration window of auditory nerve fibers upon which computational modeling of phase locking noise was based. Training on short tones resulted in improved FD on probe tests of both the long and short tones. Training on long tones resulted in improvement only on the long tones. Simulations of FD learning, based on the computational model and on signal detection theory, were compared with the behavioral FD data. We found that improved fidelity of phase locking accurately predicted transfer of learning from short to long tones, but also predicted transfer from long to short tones. The observed lack of transfer from long to short tones suggests the involvement of a second mechanism. Training may have increased the temporal integration window which could not transfer because integration time for the short tone is limited by its duration. Current learning models assume complex relationships between neural populations that represent the trained stimuli. In contrast, we propose that training-induced enhancement of the signal-to-noise ratio offers a parsimonious explanation of learning and transfer that easily accounts for asymmetric transfer of learning.

## Introduction

Perceptual learning is a long-lasting improvement in the perception of a stimulus due to experience or training. Despite much research, we are still far from a consensus on exactly what is being learned and, by extension, the neural mechanisms of that learning. Given that most training tasks are based on simple laboratory tests, the learning observed on the trained task itself, while interesting from a theoretical point of view, is often of less practical benefit than the transfer (or generalization) of learning to untrained tasks or stimuli. Understanding the rules and mechanisms of transfer and, conversely, specificity is important both for constraining hypotheses of what is being learned and for incorporating perceptual training into applications designed to improve sensory-perceptual and cognitive processing in aging or impaired populations.

Early research in visual perceptual learning has revealed an almost ubiquitous specificity of learning to the trained stimulus parameters such as retinal position or orientation (e.g., Karni and Sagi, 1991; Schoups et al., 1995). Although later studies showed specificity can sometimes be overcome (e.g., Webb et al., 2007; Xiao et al., 2008), current learning models predict transfer only between stimuli or tasks sharing access to the same processing substrates or resources (e.g., Fahle, 1994; Ahissar and Hochstein, 2004; see also Wright and Zhang, 2009 for a review of transfer in auditory learning). A major shortcoming of these models is that, in order to explain a growing number of observations of asymmetric transfer of learning, they have to resort to complex relationships between the neural populations involved in processing, such as unidirectional connections or nested populations (e.g., Matthews et al., 1999; Mossbridge et al., 2008).

Dosher and Lu (2005) have recently suggested that learning involves improving the signal-to-noise ratio (SNR) by removing the limitation on performance imposed by either external or internal noise sources (see also Gold et al., 1999). Dosher and Lu found that training with visual displays containing no added external noise transferred to noisy displays but not *vice versa*. They suggested that, in the clear displays, the processing of the target stimulus is enhanced by reducing the internal noise associated with its representation. Once improved, the benefit transfers to processing the same target stimulus in the noisy display. On the other hand, in the noisy displays, learning involves reducing the effects of the noise, a benefit that cannot transfer to a display without noise. An advantage of this model over the ones described above in explaining asymmetric transfer is that one need not assume an asymmetric neural architecture, only the ability to identify the performance-limiting source of noise.

We have recently shown that frequency discrimination (FD) learning is associated with a reduction in internal noise (Jones et al., in press), though the source of noise was not explicitly defined. Up to ∼4 kHz frequency representations are generally considered to rely on a temporal code wherein the firing of auditory nerve neurons is synchronized (phase-locked) with the periodic structure of the incoming acoustical waveforms (Moore and Glasberg, 1989). The frequency of the sound wave can be calculated from the average interval between neuronal firing over a period of time (the integration time window). Phase locking is a noisy process due to jitter in neuronal firing (Javel and Viemeister, 2000). The longer the integration time window the more accurate the frequency estimate based on the phase-locked signals because the noise cancels out. Two mechanisms could thus improve the fidelity of low frequency representations. The first is increasing the integration time window allowing averaging of the phase-locked signals over more cycles (de Cheveigne, 2005), a process limited by stimulus duration (the number of cycles actually available for averaging). The second is reducing the noise associated with phase locking directly by reducing the jitter, a process which is duration independent. Although the end result of both mechanisms is improved FD through reduction in the SNR associated with phase locking, the mechanism by which this end is achieved through training should be duration dependent.

Based on this reasoning, we hypothesized that if training on FD of long and short tones results in reducing the noise of phase locking by the same mechanism, we should see similar improvements in FD and transfer between both tone durations. However, asymmetric transfer of training would suggest the involvement of both mechanisms. As above, reduced noise of phase locking would support transfer from short to long tones. But increased integration time would be specific to the long tones so long as the window of temporal integration exceeded the duration of the short tone.

## Behavioral Data: FD Training with Long and Short Tones

### Materials and Methods

#### Participants

Forty-six adults aged 18–39 were recruited via posters from the Nottingham University student population and from the general public. They were paid an inconvenience allowance for their participation. All participants had normal hearing (pure-tone thresholds < = 20 dB HL across 0.5–4 kHz) and had no prior experience of psychoacoustic testing.

#### Ethics statement

The research protocol was approved by the Nottingham University Hospitals Research Ethics Committee. Written informed consent was obtained from all participants.

#### General procedure

**Figure 1:**
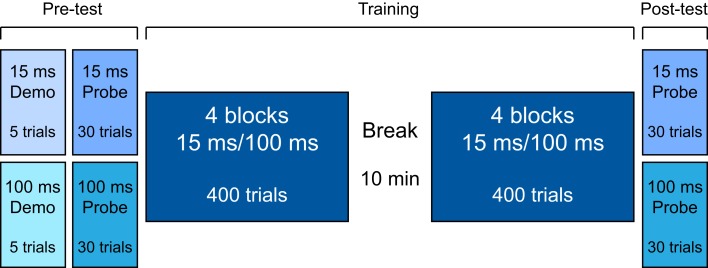
**Experimental design**.

The study protocol consisted of a pre-test phase, a training phase, and a post-test phase (Figure 1). All testing was completed within one session in a double-walled sound-attenuating booth. In all phases testing was administered via computer games with a visual interface that both cued sound presentation and provided trial-by-trial feedback for correct responses. The responses were recorded via a touchscreen. There was no time limit in which to respond, and the initiation of each trial was self-paced.

#### Stimuli

Stimuli for both training and testing consisted of 15- or 100-ms tones (including 5- or 10-ms raised cosine ramps, respectively). These stimulus durations were chosen because they are well below and above the generally accepted integration time window width of 40–50 ms (Moore, 1973). Stimuli were presented diotically at 60 dB SPL using Sennheiser HD-25-1 headphones. The frequency of the standard tone was 1000 Hz and the frequency of the target tone was adaptively varied between 1500 and 1000 Hz.

#### Pre- and post-test phases

During the pre-test and the post-test, two FD “probes” of 30 trials each were administered to every listener (Figure 1). In one probe the tones were 100-ms long (the “100-ms probe”), and in the other the tones were 15-ms long (the “15-ms probe”). The order of the probes in both pre- and post-test was counterbalanced across participants and matched between training groups. In each trial, listeners were presented with three intervals separated by a 500-ms ISI. Two intervals contained the standard tone, and the third, randomly determined interval contained a higher-frequency tone (1000 Hz + Δ*F*). They were instructed to indicate the interval that they believed was of a different pitch. Before each probe in the pre-test phase, a 5-trial demonstration was administered to familiarize participants with task requirements. Three of these “demo” trials were easy (Δ*F* = 500 Hz), and two were impossible (Δ*F* = 0 Hz). All participants correctly identified the target sounds for the Δ*F* = 500 Hz trials.

The probes used an adaptive three-down, one-up staircase procedure, targeting 79.4% correct on the psychometric function (Levitt, 1971). Δ*F* varied adaptively according to the following rule: starting with Δ*F* = 500 Hz (i.e., a target tone of 1500 Hz) it was divided by two following every correct response until the first incorrect response. Thereafter, Δ*F* was divided by √2 after three correct responses, and multiplied by √2 after one incorrect response. Difference limens for frequency (DLFs) were calculated as the 79.4% correct point on the logistic psychometric function fitted to the 30 trials in each probe, using the Wichmann and Hill (2001) optimization procedure.

Listeners were pseudo-randomly allocated to one of two training groups so as to match the two groups as closely as possible on pre-test DLFs on the 100-ms probe. Before data analysis, the two groups were carefully matched on a subject-by-subject basis after removing all listeners for whom a DLF could not be reliably established and those with outlying DLFs on the 100-ms pre-test (both low and high) so as to minimize differences due to starting thresholds. This procedure left 16 listeners in each group. The matching procedure resulted in a non-significant difference between the two groups on the 15-ms probes (*t*_30_ = 1.0; *p* = 0.33).

#### Training phase

One group trained with 100-ms tones (the “T100” group), and the other trained with 15-ms tones (the “T15” group). All listeners completed eight training blocks of 100 trials each with a 10-min rest period following the 4th training block (see Figure 1). Each training block had two interleaved tracks of 50 trials. Each track followed the same adaptive rule described above for the probes.

#### Data analysis

Difference limens for frequency (DLFs) obtained for each probe were log-transformed, resulting in normal distributions with equal variance. DLFs were analyzed using a repeated-measures ANOVA with group (T100 vs. T15) as the between-subject factor and tone duration (100 vs. 15 ms) and test (Pre-test vs. Post-test) as the within-subject factors. A “learning index” was calculated as the difference between the pre- and post-test log-transformed DLFs for each individual – positive values signify improvement on the task. The learning index for the trained task is referred to as “learning” and for the untrained task as “transfer.” The learning indices were also compared in the two groups using a repeated-measures ANOVA with group as the between-subject factor and test (or task) as the within-subject factors.

### Results

#### Training and transfer

**Figure 2:**
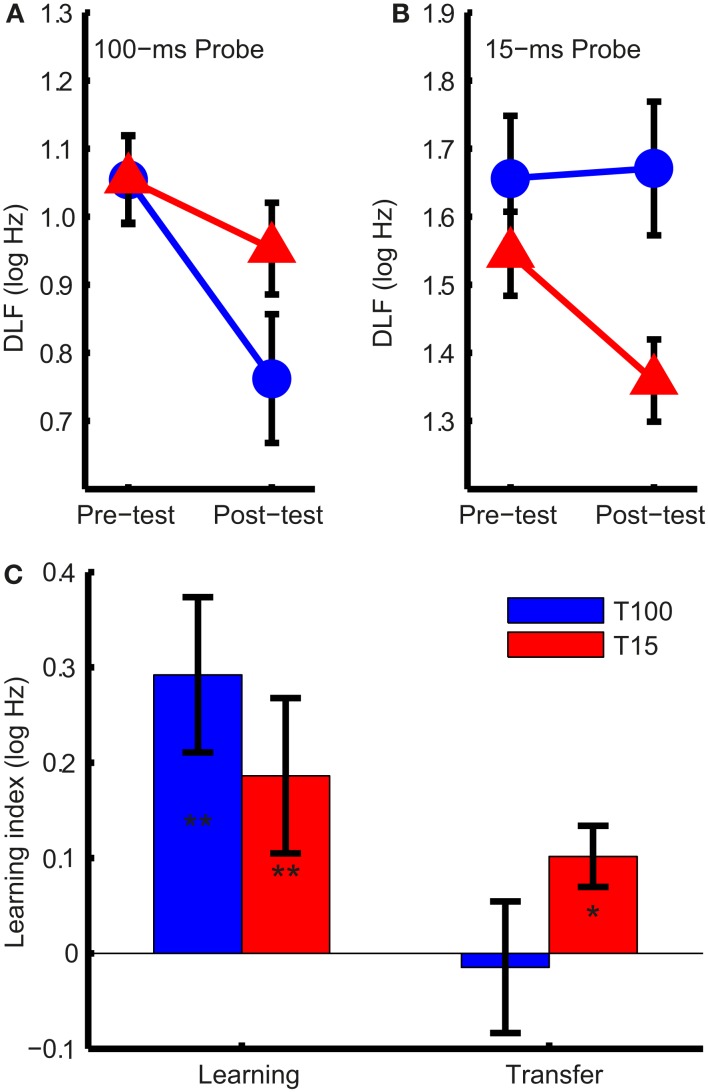
**Difference limens for frequency (DLFs) and learning indices**. **(A)** Group mean DLFs for 100-ms and **(B)** 15-ms pre- and post-test probes in the 100-ms (T100, in blue) and 15-ms (T15, in red) training groups. Note that the scales in **(A)** and **(B)** are different. **(C)** Mean group learning indices (the difference between the pre- and post-test DLFs, in log Hz) for the trained and untrained condition. Significant changes are marked with asterisks (*p < 0.05; **p < 0.01). Error bars indicate ±1 SEM. across listeners.

Difference limens for frequency for 100- and 15-ms tones are shown in Figures 2A,B, respectively. As expected (Moore, 1973), DLFs were higher for 15-ms tones (*F*_1,30_ = 217, *p* < 0.001). Both groups improved from pre- to post-test (*F*_1,30_ = 19.4, *p* < 0.001) but, critically, the change in DLFs was different in the two groups depending on tone duration (*F*_1,30_ = 7.1, *p* = 0.012). This interaction resulted from an asymmetric transfer of learning; only in the group trained on short tones did the training transfer to the untrained tone duration (Figure 2C). Exploring this interaction we found training on 100-ms tones resulted in significant learning (*t*_15_ = 3.6, *p* = 0.003) but no transfer (*t*_15_ = −0.21, *p* = 0.84), and training on 15-ms tones resulted in both significant learning (*t*_15_ = 3.2, *p* = 0.006) and transfer (*t*_15_ = 2.76, *p* = 0.037).

**Figure 3:**
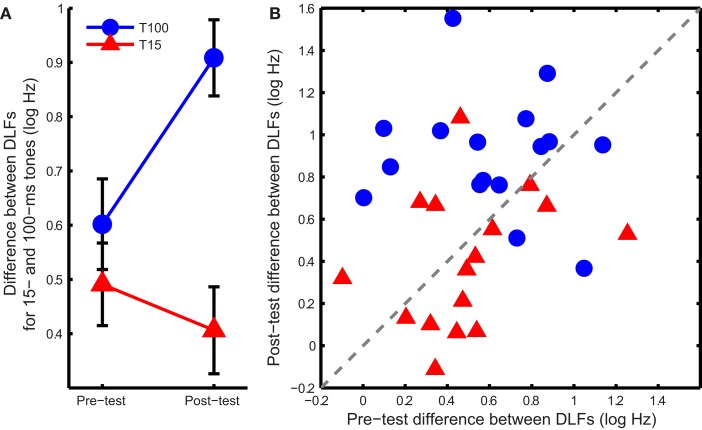
**DLF differences between long and short tones**. **(A)** Mean (±SEM) difference in DLF for 15- and 100-ms tones at pre- and post-test in the two training groups. **(B)** Individual differences between the two tone durations at pre-and post-test. Points above the dashed gray line show an increase in the difference between DLFs while points below it show a decrease.

If the learning mechanism was the same in both groups, training should affect the DLFs for short and long tones similarly. Figure 3 shows this was not the case. Training on 100-ms tones increased the difference between 15- and 100-ms DLFs while training on 15-ms tones reduced it (Figure 3A; ANOVA group effect: *F*_1,30_ = 14.1, *p* < 0.001; interaction: *F*_1,30_ = 7.1, *p* = 0.012). Most of the listeners in the T100 group showed an increase in the difference (Figure 3B), suggesting growing specificity to the 100 ms tones, while most of the listeners in the T15 group showed a reduction in the difference, suggesting increased common processing.

## Modeling Reduction in Phase Locking Noise

### Materials and Methods

We tested the hypothesis that FD learning of long and short tones involves different mechanisms, based on a model of temporal frequency coding in the auditory nerve described by de Cheveigne (2005) and on Signal Detection Theory (SDT; Macmillan and Creelman, 2005).

#### Noisy phase locking model

**Figure 4:**
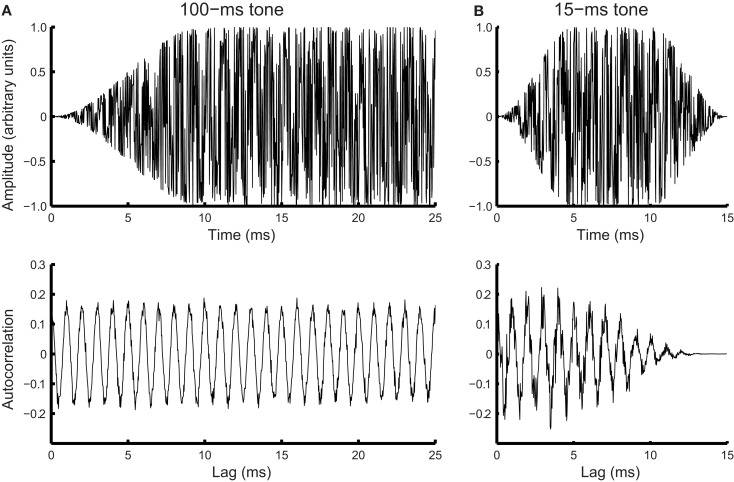
**Simulated phase locking noise**. Simulated waveform (top) and corresponding autocorrelation function (bottom) for a **(A)** noisy 100-ms, 1-kHz tone (first 25 ms of the signal shown), and **(B)** noisy 15-ms tone of the same frequency.

**Figure 5:**
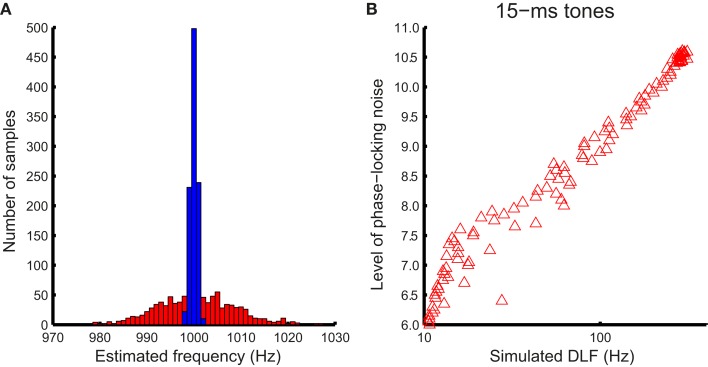
**Simulating the relationship between DLFs and phase locking noise**. **(A)** An example of estimated frequency distribution with phase locking noise. The distribution of estimated frequency for an example noise level (8.8) and the maximum integration window (50 ms) for the 100-ms 1-kHz tone (blue bars), and the entire stimulus for the 15-ms tone (red bars), obtained with 1000 iterations each. **(B)** The width (standard deviation) of the distribution was used to derive an estimated DLF for each noise level using SDT (in this case for 15-ms tones).

Noise in phase locking was simulated by a Gaussian noise added to the time domain of tonal signals (Figure 4A, top panel). Frequency of the noisy input was estimated using the autocorrelation function.

$$R\left(m\right)=\frac{1}{\left(N-m\right)}\overset{N-m+1}{\underset{n=1}{\sum }}x\left(n\right)x\left(n+m-1\right)$$

where *x*(*n*) is the noise-laden tonal signal (in the form of a time series), *n* is the temporal order of the data points, *m* is the lag, and *N* is the length of the time series. The autocorrelation function was weighted by the duration of the sample used (biased), so that the peaks at different lags were of comparable amplitude to facilitate peak identification (Figure 4A, bottom panel). The temporal positions of the peaks of the autocorrelation function were averaged to yield a frequency estimate. For the 15-ms tone (Figure 4B), the integration time window was the entire stimulus. The simulated noise covered a large range determined by initial pilot simulations. For each simulated noise level 1000 iterations yielded a distribution of frequency estimates based on the autocorrelations functions [see Figure 5A for an example of frequency distributions for 100- (blue) and 15-ms tones (red) for a single noise level]. The means of the distributions matched the input signal frequency (1 kHz, SE < 0.001 Hz for 1000 iterations) for the entire noise range used. The standard deviation of the distribution was then used to simulate the DLFs for 100- and 15-ms tones according to SDT. For the 3-interval forced-choice paradigm used in the experiment, DLFs were estimated at 79% correct, i.e., thresholds were estimated to be ∼1.6 times the standard deviation of the frequency sensation distribution. The estimated DLFs as a function of noise level are shown in Figure 5B for the 15-ms tones.

#### Estimating the phase locking noise in the behavioral data

Phase locking noise at pre-test was estimated for each participant by matching the behavioral pre-training DLFs to the predicted noise using the function estimated in Figure 5B. Under the assumption that the internal noise associated with phase locking would be the same for both tone durations in naïve listeners because phase locking jitter is an intrinsic property of neurons, we derived the integration time for 100-ms tones that would yield the observed DLFs.

### Results

#### Learning and transfer of reduction in phase locking noise

**Figure 6:**
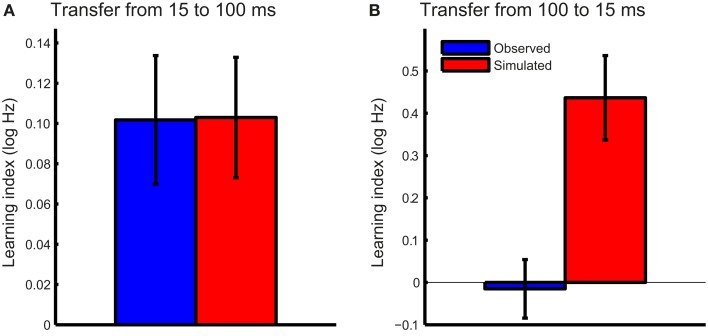
**Observed and model-predicted transfer**. The mean observed transfer (blue bars) and the simulated transfer predicted by the model based on improved phase locking (red bars) were plotted for **(A)** the T15 and **(B)** the T100 training groups. Error bars indicate ±1 SEM.

To test the hypothesis that FD learning for short tones was consistent with improved phase locking, we first tested whether this mechanism could account for the transfer from 15- to 100-ms tones in the T15 group. The noise levels associated with the observed DLFs on the trained (15 ms) condition were estimated for the pre- and post-test DLFs using the function illustrated in Figure 5B. Change in phase locking noise was taken as the difference between the pre- and post-test noise estimates. Expected post-test DLFs in the untrained 100-ms condition were then calculated with the integration time derived from pre-test DLFs under the assumption that phase locking properties are stimulus-independent, and thus the improvement in phase locking noise transferred fully between conditions. The learning index was calculated as the difference between estimated post-test DLFs and observed pre-test DLFs for 100-ms tones (Figure 6A). The modeled learning index accurately predicted the transfer from 15- to 100-ms tones in the group trained on short tones (two-tailed *t*-test: *t*_15_ = −0.03, *p* = 0.98).

We then tested the hypothesis that training on 100-ms tones is similarly related to a reduction in phase locking noise. The same procedure described above was repeated for the T100 group, with changes in phase locking noise estimated based on the observed pre-and post-test DLFs and estimated individual integration times. The modeled transfer to the 15-ms tones under the same assumption of full transfer of phase locking noise reduction far exceeded that observed in this group (Figure 6B; *t*_15_ = −3.4, *p* = 0.004). If training on 100-ms tones reduced phase locking noise, we would have expected much greater transfer to the 15-ms condition.

#### Transfer from 100- to 15-ms tones

We can conclude from the above simulation that the same mechanisms for reducing the phase locking noise cannot explain the observed transfer results in both training groups, suggesting different mechanisms may be at work in reducing the noise in frequency representation. As we have argued in the Introduction, noise in the 100-ms tones can be reduced by increasing the integration time window. Indeed, we estimated the pre-test integration time for the 100-ms tones at 16.6 ± 9.2 ms (mean ± SD), which is longer than the integration time for 15-ms tones that include 5-ms rise- and fall ramps and results in the pre-test difference in DLFs between the two condition. Moore (1973) has measured the integration time window beyond which FD in well-trained listeners no longer improve with increased tone duration at ∼50 ms, suggesting training does increase the integration time. Since the naïve integration time already exceeds the duration of the short tones, no transfer is expected for this mechanism, as we have observed.

## Discussion

We demonstrate here an asymmetric transfer of learning between (task-irrelevant) tone durations on a FD task. Training on long tones resulted in no transfer to short tones whereas training on short tones showed transfer. We simulated FD learning as a reduction in phase locking noise and showed that whereas transfer from short to long tones is accurately predicted by this computational model, it cannot explain the lack of transfer from long to short tones. Thus, training on the long tones must have modified a different mechanism. These results provide a parsimonious explanation of asymmetric transfer without the need to resort to complex neural architectures.

The effect of duration on pure-tone FD learning and transfer has been previously investigated by Delhommeau et al. (2002). They found training on 200-ms tones transferred fully to 100-ms tones but only partially to 40-ms tones, and suggested two alternative explanations. Firstly, long (e.g., 200, 100 ms) and short (e.g., 40 ms) tones are differentially encoded by different mechanisms: a temporal code based on timing of neuronal firing in the auditory nerve for the longer tones, and a place code based on the activated location on the cochlea for the shorter tones where the paucity of cycles used in phase locking (short available integration time) reduces the fidelity of the frequency representation. However, it is widely accepted that the primary encoding mechanism for frequencies below 4–5 kHz is temporal rather than a place code regardless of duration (Moore, 1973). Alternatively, they suggested that long and short tones activate partially overlapping neuronal populations, with the population encoding 100-ms tones having greater overlap with the population encoding 200-ms tones than those encoding 40-ms tones. Critically, Delhommeau and colleagues did not test transfer in the opposite direction. We suggest that the observed transfer seen in their experiment may have resulted from an increased integration time window induced by training on the long tones, which transferred fully to tones longer than the post-training time widow (100 ms) but only partially to shorter tones because the 40-ms tone duration presented an upper bound on window width. Compared to our 15-ms tones, it was still long enough to show some benefit of transfer (based on our simulation we estimated the lower bound of integration time in naïve listeners to be ∼15 ms long, see Section “Transfer from 100- to 15-ms tones”).

Physiological data lend further support to the proposed mechanism for FD learning by improved phase locking (Carcagno and Plack, 2010). Carcagno and Plack show that training on FD improves the synchronization of the frequency-following response (FFR) to the envelope of the presented sound. The FFR is an auditory evoked potential thought to originate in the auditory brainstem, and reflects neural phase locking to incoming sounds. The process they describe could reflect a reduction in phase locking noise through reduced jitter in neuronal firing, which in turn could depend on training-task-specific attentional gating of signals via the descending pathways of the efferent auditory system (de Boer and Thornton, 2008). Although the phase locking mechanism is generally considered low-level and depending on bottom-up inputs, training-induced changes can be driven top-down by a high-level process such as attention.

We propose that the mechanisms described above for learning in FD are a specific instance of a more general model of learning as an increase in the SNR by the gradual removal of performance-limiting noise sources during the training process. This model bypasses the distinction between bottom-up and top-down influences on learning since the internal noise limiting psychophysical performance can originate on many different levels: neuronal (e.g., stochastic firing: Javel and Viemeister, 2000), systemic (e.g., heartbeat or blood flow: Soderquist and Lindsey, 1971), or cognitive (e.g., fluctuations in attention: Faisal et al., 2008). The controversy of whether learning is a top-down or bottom-up phenomenon is therefore replaced by a single principle of determining the source of performance-limiting noise. Moreover, it eliminates the distinction between stimulus- and “non-stimulus” learning previously described as procedural (Robinson and Summerfield, 1996; Hawkey et al., 2004; Ortiz and Wright, 2009), conceptual (Wright and Zhang, 2009; Ortiz and Wright, 2010), or strategy learning (Pellegrino et al., 1991; Doane et al., 1996, 1999). Any aspect of the stimulus, task or procedure could introduce noise that affects performance thresholds and is potentially subject to training-induced reduction. One implication of this model is that long-term training may reduce multiple types of noise over time. As one source of performance-limiting noise is reduced, other sources may become more prominent in constraining performance. For example, an initial, rapid reduction in noise associated with choosing the correct motor response may occur before the reduction of noise that constrains stimulus encoding, contributing to the extremely rapid early improvement followed by slower learning often seen in learning curves (Robinson and Summerfield, 1996; Hawkey et al., 2004).

It is worth mentioning here that this model is compatible with a revised version of the reverse hierarchy theory of visual learning (RHT; Ahissar and Hochstein, 2004). This theory originally postulated that learning occurs at the highest level in the processing hierarchy capable of carrying out the training task, with the search for this level proceeding under attentional control in a high- to low-level direction. We propose here that attention can guide the search for the mechanism that will reduce or eliminate performance-limiting noise rather than a representational level and that in vision this search would ideally be carried out in reverse along the hierarchy. However, since much of the processing of specific sound features (such as frequency and location) in the auditory system is subcortical, it might be preferable to eliminate low-level sources of noise early on in this modality (see Amitay, 2009).

## Conflict of Interest Statement

The authors declare that the research was conducted in the absence of any commercial or financial relationships that could be construed as a potential conflict of interest.

## References

[B1] Ahissar M. Hochstein S. (2004). The reverse hierarchy theory of visual perceptual learning. Trends Cogn. Sci. (Regul. Ed.) 8, 457–464 10.1016/j.tics.2004.08.011 15450510

[B2] Amitay S. (2009). Forward and reverse hierarchies in auditory perceptual learning. Learn. Percept. 1, 59–68 10.1556/LP.1.2009.1.5

[B3] Carcagno S. Plack C. (2010). Subcortical plasticity following perceptual learning in a pitch discrimination task. Invest. Opthalmol. Vis. Sci. 12, 89–100 10.1007/s10162-010-0236-1 PMC3015031 20878201

[B4] de Boer J. Thornton A. R. D. (2008). Neural correlates of perceptual learning in the auditory brainstem: efferent activity predicts and reflects improvement at a speech-in-noise discrimination task. J. Neurosci. 28, 4929–4937 10.1523/JNEUROSCI.0902-08.2008 18463246 PMC6670751

[B5] de Cheveigne A. (2005). “Pitch perception models,” in Pitch: Neural Coding and Perception, eds Plack C. J. Oxenham A. J. Fay R. R. Popper A. N. (New York: Springer), 169–233

[B6] Delhommeau K. Micheyl C. Jouvent R. Collet L. (2002). Transfer of learning across durations and ears in auditory frequency discrimination. Percept. Psychophys. 64, 426–436 10.3758/BF03194715 12049283

[B7] Doane S. M. Alderton D. L. Sohn Y. W. Pellegrino J. W. (1996). Acquisition and transfer of skilled performance: are visual discrimination skills stimulus specific? J. Exp. Psychol. Hum. Percept. Perform. 22, 1218–1248 10.1037/0096-1523.22.5.1218

[B8] Doane S. M. Sohn Y. W. Schreiber B. (1999). The role of processing strategies in the acquisition and transfer of a cognitive skill. J. Exp. Psychol. Hum. Percept. Perform. 25, 1390–1410 10.1037/0096-1523.25.5.1390

[B9] Dosher B. A. Lu Z. L. (2005). Perceptual learning in clear displays optimizes perceptual expertise: learning the limiting process. Proc. Natl. Acad. Sci. U.S.A. 102, 5286–5290 10.1073/pnas.0500492102 15795377 PMC555685

[B10] Fahle M. (1994). Human pattern recognition: parallel processing and perceptual learning. Perception 23, 411–427 10.1068/p230411 7991342

[B11] Faisal A. A. Selen L. P. J. Wolpert D. M. (2008). Noise in the nervous system. Nat. Rev. Neurosci. 9, 292–303 10.1038/nrn2258 18319728 PMC2631351

[B12] Gold J. Bennett P. J. Sekuler A. B. (1999). Signal but not noise changes with perceptual learning. Nature 402, 176–178 10.1038/46027 10647007

[B13] Hawkey D. J. Amitay S. Moore D. R. (2004). Early and rapid perceptual learning. Nat. Neurosci. 7, 1055–1056 10.1038/nn1315 15361880

[B14] Javel E. Viemeister N. F. (2000). Stochastic properties of cat auditory nerve responses to electric and acoustic stimuli and application to intensity discrimination. J. Acoust. Soc. Am. 107, 908–921 10.1121/1.428269 10687700

[B15] Jones P. R. Shub D. E. Moore D. R. Amitay S. (in press). Reduction of internal noise in auditory perceptual learning. J. Acoust. Soc. Am. 10.1121/1.4773864 23363114

[B16] Karni A. Sagi D. (1991). Where practice makes perfect in texture-discrimination: evidence for primary visual-cortex plasticity. Proc. Natl. Acad. Sci. U.S.A. 88, 4966–4970 10.1073/pnas.88.11.4966 2052578 PMC51788

[B17] Levitt H. (1971). Transformed up-down methods in psychoacoustics. J. Acoust. Soc. Am. 49, 467–477 10.1121/1.1912375 5541744

[B18] Macmillan N. A. Creelman C. D. (2005). Detection Theory: A User’s Guide. Mahwah, NJ: Lawrence Erlbaum Associates, Inc

[B19] Matthews N. Liu Z. L. Geesaman B. J. Qian N. (1999). Perceptual learning on orientation and direction discrimination. Vision Res. 39, 3692–3701 10.1016/S0042-6989(98)00249-1 10746139

[B20] Moore B. C. J. (1973). Frequency difference limens for short-duration tones. J. Acoust. Soc. Am. 54, 610–619 10.1121/1.1914343 4754385

[B21] Moore B. C. J. Glasberg B. R. (1989). Mechanisms underlying the frequency discrimination of pulsed tones and the detection of frequency modulation. J. Acoust. Soc. Am. 86, 1722–1732 10.1121/1.398086

[B22] Mossbridge J. A. Scissors B. N. Wright B. A. (2008). Learning and generalization on asynchrony and order tasks at sound offset: implications for underlying neural circuitry. Learn. Mem. 15, 13–20 10.1101/lm.573608 18174369 PMC2170511

[B23] Ortiz J. A. Wright B. A. (2009). Contributions of procedure and stimulus learning to early, rapid perceptual improvements. J. Exp. Psychol. Hum. Percept. Perform. 35, 188–194 10.1037/a0013161 19170481 PMC2866737

[B24] Ortiz J. A. Wright B. A. (2010). Differential rates of consolidation of conceptual and stimulus learning following training on an auditory skill. Exp. Brain Res. 201, 441–451 10.1007/s00221-009-2053-5 19902196 PMC2967583

[B25] Pellegrino J. W. Fischer S. C. Doane S. M. Alderton D. (1991). Stimulus complexity effects in visual comparisons: the effects of practice and learning context. J. Exp. Psychol. Hum. Percept. Perform. 17, 781–791 10.1037/0096-1523.17.3.781 1834790

[B26] Robinson K. Summerfield A. Q. (1996). Adult auditory learning and training. Ear Hear. 17, 51S–65S 10.1097/00003446-199617031-00006 8807276

[B27] Schoups A. A. Vogels R. Orban G. A. (1995). Human perceptual learning in identifying the oblique orientation: retinotopy, orientation specificity and monocularity. J. Physiol. (Lond.) 483, 797–810 7776259 10.1113/jphysiol.1995.sp020623 PMC1157819

[B28] Soderquist D. R. Lindsey J. W. (1971). Physiological noise as a low-frequency masker: the cardiac cycle. J. Acoust. Soc. Am. 50, 143 10.1121/1.1977681 4638036

[B29] Webb B. S. Roach N. W. Mcgraw P. V. (2007). Perceptual learning in the absence of task or stimulus specificity. PLoS ONE 2, e1323 10.1371/journal.pone.0001323 18094748 PMC2147046

[B30] Wichmann F. A. Hill N. J. (2001). The psychometric function: I. Fitting, sampling, and goodness of fit. Percept. Psychophys. 63, 1293–1313 10.3758/BF03194544 11800458

[B31] Wright B. A. Zhang Y. (2009). A review of the generalization of auditory learning. Philos. Trans. R. Soc. Lond. B Biol. Sci. 364, 301–311 10.1098/rstb.2008.0262 18977731 PMC2674478

[B32] Xiao L. Q. Zhang J. Y. Wang R. Klein S. A. Levi D. M. Yu C. (2008). Complete transfer of perceptual learning across retinal locations enabled by double training. Curr. Biol. 18, 1922–1926 10.1016/j.cub.2008.10.030 19062277 PMC3045109

